# ZnO nanocrystals shuttled by extracellular vesicles as effective Trojan nano-horses against cancer cells

**DOI:** 10.2217/nnm-2019-0231

**Published:** 2019-11-21

**Authors:** Bianca Dumontel, Francesca Susa, Tania Limongi, Marta Canta, Luisa Racca, Angelica Chiodoni, Nadia Garino, Giulia Chiabotto, Maria L. Centomo, Ymera Pignochino, Valentina Cauda

**Affiliations:** 1 Department of Applied Science & Technology, Politecnico di Torino, Corso Duca degli Abruzzi 24, 10129 Turin, Italy; 2 Center for Sustainable Future Technologies – CSFT@POLITO, Istituto Italiano di Tecnologia, Via Livorno, 60, 10144 Turin, Italy; 3 Sarcoma Unit, Division of Medical Oncology, Candiolo Cancer Institute, FPO – IRCCS, Str. Prov.le 142, km. 3.95, Candiolo (TO) 10060, Italy; 4 Department of Medical Sciences, University of Torino, Torino 10126, Italy; 5 Department of Oncology, University of Torino, Str. Prov.le 142, km. 3.95, Candiolo (TO) 10060, Italy

**Keywords:** biomimetics, colloidal stability, cytotoxicity, extracellular vesicles, nanocrystals, zinc oxide

## Abstract

The effective application of nanoparticles in cancer theranostics is jeopardized by their aggregation in biological media, rapid degradation and clearance. The design of biomimetic nanoconstructs with enhanced colloidal stability and non-immunogenicity is therefore essential. We propose naturally stable cell-derived extracellular vesicles to encapsulate zinc oxide (ZnO) nanocrystals as efficacious nanodrugs, to obtain highly biomimetic and stable Trojan nano-horses (TNHs).

**Materials & methods:**

Coupling efficiency, biostability, cellular cytotoxicity and internalization were tested.

**Results:**

*In vitro* studies showed a high internalization of TNHs into cancer cells and efficient cytotoxic activity thanks to ZnO intracellular release.

**Conclusion:**

TNHs represent an efficient biomimetic platform for future nanotheranostic applications, with biomimetic extracellular vesicle-lipid envelope, facilitated ZnO cellular uptake and potential therapeutic implications.

## Graphical abstract

**Graphic abstract:**
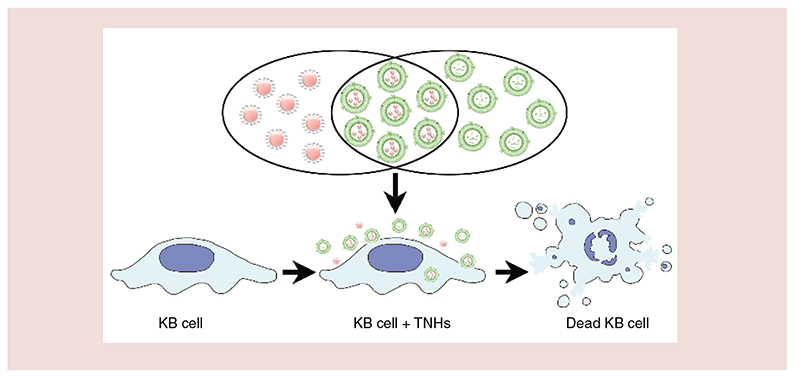

In the last few years, a wide range of customizable therapeutic nanoparticles (NPs) have been explored for biotechnological applications in biological and medical contexts [1,2]. These kinds of particles should be designed as biomimetic tools [3], able to mimic biological function and to modulate cell role by imitating features of the systems and surfaces that they are designed to engage [4]. The use of biomimetic NPs, with enhanced colloidal stability and dispersion in biological and human fluids, can avoid side effects, like rapid clearance and unspecific uptake [5,6]. For the design of biocompatible NPs with a high level of biomimicry, several important factors should be considered, such as the designation of the core material, the shape and surface chemistry. In the last decades, two different bioengineering and nanotechnology approaches have been used to optimize the design of biomimetic smart tools mimicking cell surface’s biochemistry and morphology. The bottom-up approach, to match or imitate cells physiopathological features, refers to process as surface conjugation [7,8], protein linkage [9] and/or lipid coating [10–12]. On the other hand, instead of producing an artificial membrane, top-down approaches directly apply biological components to the particle cores [4]. For example, to customize more effective biocompatible NPs, many researchers have just isolated different kinds of biological membranes from cells and subcellular compartments to coat metallic or polymeric cores. Red blood cells, leukocytes, platelets or mesenchymal stem cells have been used for the production of cellular ‘ghosts’ that encapsulate NPs and thus increase their biomimicry [13,14].

In this context, recent interest has raised on extracellular vesicles (EVs), in other words, cell-derived lipid membrane particles, secreted by various cell types including normal and diseased cells (i.e., cancer cells) [15]. EVs can be isolated from many physiological fluids or conditioned cell culture media [16]. The term EVs indicates several vesicle populations, which differ in size, molecular composition and biogenesis. Exosomes are vesicles of endocytic origin with a diameter of 30–100 nm that are released extracellularly after the multivesicular bodies fuse with the plasma membrane. In contrast, the microvesicles are slightly greater in size (100–500 nm) and bud directly from the plasma membrane [15]. Exosomes and microvesicles have demonstrated different biological functions and thus express different molecular markers on their surface [15]. However, a complete separation and purification of the two types of vesicles, either based on their size or on their immune-affinity, is extremely difficult, at least to date. Therefore, many investigations, especially in the field of drug delivery, utilize both exosomes and microvesicles, in other words, EVs [17]. Actually, the natural role of EVs, responsible for intercellular communication, provides them with fundamental characteristics, exploitable for the formulation of drug delivery vehicles, like natural stability in blood and other biological fluids, and an intrinsic ability to cross biological barriers [18,19]. Furthermore, it was suggested that allogenic exosomes, collected from patients’ tissues and blood, should benefit from an immune-privileged status with low clearance by mononuclear phagocyte system and prolonged blood circulation [18]. Additionally, some studies indicate that EVs molecular composition, which reflects their parental cell source, may confer specific cell or tissue tropism, determining their biodistribution and possible intrinsic homing properties [20]. Thanks to these unique characteristics together with their biocompatibility, EVs have recently been considered as drug and gene delivery vehicles for low-molecular-weight therapeutics and nucleic acids [21].

Comparing EVs with traditional polymer-based synthetic NPs, NPs alone can show high toxicity and lack of targeting specificity, while it was found that membrane vesicles fused more effectively with the plasma membrane of cancer cells and enabled the codelivery of hydrophobic and hydrophilic compounds, largely bypassing the endosome/lysosome pathway and thus enhancing the delivery of the cargo in a biologically active form [22]. Very recent studies in the context of cancer treatment report the combination of synthetic NPs (gold NPs and metalorganic framework) with EVs, in order to exploit the advantageous features of both components. In those studies, the stabilization and biomimicry provided by exosome shielding is combined with the imaging properties [23] and the high drug-loading ability [24,25] of traditional nanocarriers.

In the present work, we analyze the possibility to combine EVs with zinc oxide nanocrystals (ZnO NCs). Unlike gold and metal-organic framework NPs that elicit a biological response thanks to a loaded chemotherapeutic agent, ZnO NCs have intrinsic cytotoxic properties above certain concentrations [26], despite when designed safe by anchoring specific ligands [27]. Even if still under debate, one of the main mechanisms for ZnO cytotoxicity is related to the intracellular dissolution and release of Zn^2+^ ions, causing mitochondrial damage and disruption of cellular zinc homeostasis. This can lead to a protein activity disequilibrium that affects several cellular processes [28]. A second cytotoxicity mechanism is connected to reactive oxygen species (ROS) production, due to either proinflammatory cell response against NPs and to characteristic surface property of ZnO NCs [28]. In fact, the semiconductor properties of ZnO make the NPs a site of redox reaction for the generation of ROS, responsible for increased oxidative stress and eventually cell death. Interestingly, many *in vitro* studies showed a preferential ZnO cytotoxicity toward cancer cells rather than for healthy ones [29], indicating its promising application and efficiency in cancer therapy. In particular, the proliferative, osteo-inductive and differentiating properties of ZnO NCs on healthy preosteoblasts were recently reported [30], thus showing the positive effect of ZnO when healthy cells are involved. However, from the clinical applications perspective, the precise control of ZnO nanomaterials biocompatibility, aggregation and interaction with components of biological media need to be carefully addressed [10,31]. Actually, several studies demonstrated the ZnO NCs reactivity toward different plasma proteins [32] and inorganic components, especially phosphate ions [33,34], which can deeply affect the immune response, biodistribution and cytotoxicity.

In this scenario, we decided to combine nonimmunogenic and naturally stable cell-derived EVs with our synthetic ZnO NCs, to enhance their colloidal stability and biomimicry, with the final aim to obtain a biomimetic platform for intracellular delivery of ZnO NCs. Using a biocompatible strategy, NCs were incorporated within EVs with an average coupling efficiency of about 40%, outdoing the combination efficiency of previous similar works. These EV-mimicking NPs were thus called Trojan nano-horses (TNHs), to convey the concept of biomimetism of the ZnO NCs shielded by the EVs membrane.

Through *in vitro* study, we demonstrated a higher biostability of our TNH NCs with respect to the pristine ZnO NCs and an efficient intracellular delivery toward cancer cells. Notably, the TNHs are internalized at a high level even when added at low concentration to cell culture media. Furthermore, we noted that the EV-derived lipids closely interact with the plasma cell membrane, releasing their therapeutic cargo, in other words, the ZnO NCs, inside the cells. Therefore, the released ZnO NCs are able to efficiently exploit their cytotoxic activity, acting as a nanodrug.

We further recall the ability of ZnO NCs, being a semiconductor material, to also work as a bioimaging agent thanks to its widely explored optical emission properties, as recently demonstrated [35]. Therefore, the ZnO NCs can act both as therapeutic and diagnostic tools in a final theranostic approach.

Differently from our previous reports focused on the ZnO NCs synthesis approaches [35] or biostabilization with commercially available lipid bilayers [10], here we propose a novel nanoconstruct showing the threefold advantage of: proving the efficacy of ZnO NCs as nanodrugs, internalized in cancer cells; stabilizing them and increasing their internalization into cancer cells thanks to the cell-derived lipid biovesicles; and gaining potential non-immunogenicity when preparing these NCs using autologous EVs from an hypothetical patient.

Our results propose a new nanotechnological strategy to construct reproducible and efficient biomimetic nanoplatforms for highly efficient intracellular delivery of cytotoxic NCs and for future nanotheranostic applications.

## Materials & methods

### ZnO NC synthesis, chemical functionalization & labeling

ZnO NCs were prepared through a novel microwave-assisted solvothermal approach [30,35]. Zinc acetate dihydrate (0.1 M) and KOH (0.2 M) (both from Sigma-Aldrich, MO, USA) were used as precursors and dissolved in two separate solutions using methanol (Reag. Ph.Eur ACS; VWR, PA, USA) as a solvent. The zinc precursor solution was stirred directly in the microwave-reactor vessel. In order to initiate the zinc oxide nucleation, 0.48 μl of double-distilled (dd) water was added and then the KOH solution was mixed together in the reactor vessel, the final solution pH was 8. The resulting solution was put into the microwave oven for 30 min under control of pressure and temperature (60°C, maximum microwave power of 150 W). After this time, the colloidal solution was centrifuged (10 min at 3500 × *g*), the supernatant was discarded and the newly formed ZnO NCs were washed twice and finally dispersed in 15 ml of ethanol (99%, Sigma-Aldrich).

The functionalization of ZnO NCs surface with amino groups (-NH_2_) was obtained by combining the NCs with 3-aminopropyltrimethoxysilane (APTMS; 97%, Sigma-Aldrich) as already reported in [10,30,35]. In details, 100 mg (1.23 mmol) of ZnO NCs dispersed in ethanol was heated to 70°C in a 50-ml round glass flask under continuous stirring and nitrogen gas flow. After 15 min, 21.4 μl of APTMS (0.123 mmol, 22.05 mg, corresponding to 10 mol% of total ZnO amount) was added to the solution. The obtained mixture was refluxed under a nitrogen gas flow for 6 h and successively washed twice by centrifuging (10,000 × *g*, 5 min) and redispersing the NCs in fresh ethanol to remove the unbound APTMS molecules.

The amine-functionalized ZnO NCs were coupled with Atto647-NHS ester (ATTO-TEC, λ_Ex_ = 643 nm) in a ratio of 2 μg/mg of NCs. The solution in ethanol was stirred in the dark overnight and then washed twice (see above, to remove unbounded dye molecules) [10].

### Cell culturing, EV extraction & labeling

KB cell line (CCL17™) was purchased by the American Type Culture Collection (ATCC®, VA, USA). Cells were grown in Minimal Essential Eagle’s Medium (Sigma-Aldrich) supplemented with 10% heat-inactivated fetal bovine serum (FBS; Sigma-Aldrich), 100 units/ml penicillin and 100 μg/ml streptomycin (Sigma-Aldrich) and maintained at 37° C under a 5% CO_2_ atmosphere. Cells were periodically tested for mycoplasma infection.

To deplete the medium containing 20% of FBS, an MLA-50 rotor of the ultracentrifuge OptiMax from Beckman Coulter with sterile polypropylene tubes (Optiseal, nominal capacity 32 ml) was used. The tubes were loaded and then centrifuged overnight (14 h) at 100,000 × *g* at 4°C. The supernatant was collected and then diluted with fresh medium up to a final concentration of 10% FBS and then used to culture KB cells for EV production and isolation. For EV extraction, cells were counted by using a TC20™-automated cell counter (BioRad Laboratories, CA, USA) and 1 × 10^6^ cells were plated in a 75 cm^2^ treated flask (Corning TC-treated) with 20 ml of complete culture medium. Nine flasks were prepared for each extraction. After 48 h culturing, cells were washed with phosphate-buffered saline (PBS) and the culture medium was replaced with 20 ml/flask of exosome-free medium. After 48 h, the EVs were extracted.

The EV extraction protocol is based on a sterile differential ultracentrifugation protocol optimized modifying the one described in [16]. Before EV isolation, cell vitality was always assessed via a Trypan-blue (VWR)-hased imaging system (TC-10, BioRad Laboratories). Only samples with a viability ≥95% were processed to reduce the possibility of recovery apoptotic bodies. The cell medium was collected into 50-ml tubes and centrifuged for 10 min at 130 × *g* at 4°C in order to remove dead cells. The supernatant was then centrifuged again for 20 min at 2000 × *g* at 4°C to remove cell debris. The supernatants were then collected again, placed into ultracentrifuge Optiseal tubes and centrifuged at 10,000 × *g* for 30 min at 4°C to remove the aggregates of biopolymers, any apoptotic bodies and the other structures with a density higher than that of EVs. The supernatants were then recollected and ultracentrifuged at 100,000 × *g* for 70 min at 4°C. The obtained pellet was resuspended in cold, sterile, 0.1-μm filtered PBS solution and centrifuged at 100,000 × *g* for further 60 min at 4°C. The pellet, which contained EVs, was then resuspended in 600 μl of sterile, cold PBS or physiologic solution (0.9% NaCl; NovaSelect, Italy) and, once aliquoted to 50 μl in cryovials, they were stored at -80°C for further use. To perform fluorescence microscopy or cytofluorimetry analyses, the EVs were labeled by using DiOC_18_(3) (3,3′-dioctadecyloxacarbocyanine perchlorate) (DiO; λ_Ex_ = 484 nm, Invitrogen, CA, USA) or 1,1′-dioctadecyl-3,3,3′,3′-tetramethylindodicarbocyanine perchlorate (DiD; λ_Ex_ = 648 nm, Invitrogen). EVs were thawed out from -80°C and labeled by adding 0.5 μl of dye solution (10 μM in DMSO) to each aliquot, containing approximately 7 × 10^9^ EVs. The obtained solution was put under agitation in dark, at 180 rpm, 37°C for 30 min and then ultracentrifuged once in PBS at 100,000 × *g*, 4°C for 60 min in order to wash out the unbound dye molecules. The obtained pellet was resuspended in cold-filtered PBS or physiologic solution.

### TNH construction

The coupling between EVs and ZnO NCs was carried out by varying several parameters, like the mixing method, the duration and the temperature of the reaction, the type of dispersing medium and the ratio between the two components. The coupling process was carried out using solutions filtered with Acrodisc syringe filter with 0.2 μm GHP membrane (Pall Corporation, NY, USA). 50 μl of fluorescently labeled EVs solution was added to a 50-μl solution of sterile ultrapure dd water (MilliQ system, Millipore, MA, USA) containing different concentrations of fluorescently labeled ZnO NCs, as required by the screening of the experimental conditions detailed below. At the end of the coupling process, the samples were centrifuged at a low centrifugation acceleration, in other words, 5000 × *g* for 5 min. The obtained pellet containing the first run of ZnO coupled to EVs (i.e., the TNH Run 1) was dispersed in 100 μl of 1:1 (v/v) of water and PBS or physiologic solution. The supernatant, containing mainly uncoupled EVs, was coincubated with new aliquots of ZnO NCs, repeating both the mixing and centrifugation processes and thus leading to further TNHs of Run 2.

The protocol described above has been amended as follows for all the biological assays to treat KB cancer cells. The concentrations of NCs used for each cell treatment were as a whole 5, 15, 25 and 50 μg/ml, calculated with respect to final volume used to treat cells. After the first run of the TNH-coupling process (TNH Run 1), the centrifuged pellet was resuspended directly in the cell culture medium (635 μl), while the supernatant (containing still empty EVs in 365 μl of mixed water and physiologic solution) was coupled with a new aliquot of ZnO NCs (TNH Run 2). At the end of this run, no centrifugation was performed and both TNH Run 1 and TNH Run 2 were reunited obtaining a final volume of 1 ml. KB cells were then treated with this TNHs solution. More details on the used amounts of ZnO and EVs for the TNH preparation step and the composition of the solution are reported in Supplementary Table 1.

For control experiments, KB cells were also treated with only the amine-functionalized ZnO NC samples or only the EV samples, following all the steps for the TNH formation, without the presence of EVs or ZnO NCs, respectively.

### Characterization methods

The amine-functionalized ZnO NCs were characterized by x-ray diffraction (XRD) with a Cu-Kα source of radiation, operating at 40 kV and 30 mA in configuration θ–2θ Bragg-Brentano (Panalytical X’Pert diffractometer, Malvern Panalytical, Malvern, UK). The samples were prepared depositing several drops of colloidal solution on a silicon wafer obtaining a sufficiently thick layer of dried NCs and the XRD spectrum was collected in the range of 20–65° with a step size of 0.02° (2θ) and an acquisition time of 100 s.

Transmission electron microscopy (TEM) images were collected by a FEI Tecnai F20 ST transmission electron microscope. For obtaining high-resolution TEM images of ZnO NCs, an accelerating voltage of 200 kV was used while for EVs and TNHs, the accelerating voltage was set at 80 kV. All the analyzed samples were diluted in ultrapure ethanol (99%) up to a concentration of 100 μg/ml for ZnO NCs or in a solution 1:1 v/v of dd water and physiologic solution for both EV and TNH. One drop of each sample was deposited on a holey carbon copper grid with 300-carbon mesh and left to dry overnight.

For field emission scanning electron microscopy (FESEM), purified EVs were diluted in 0.1-μm filtered PBS and 10-μl drops were deposited on silicon wafers. The samples were dried at room temperature (RT) and then analyzed by FESEM (Merlin, Carl Zeiss, Oberkochen, Germany).

Dynamic light scattering (DLS) and ζ-potential measurements were carried out with Zetasizer Nano ZS90 (Malvern Panalytical), measuring ZnO NCs in ethanol, water, PBS and physiologic solution at a concentration of 100 μg/ml. ζ-potential measurements of ZnO NCs, EVs and TNHs were performed with the same instrument, diluting the 50 μl of sample aliquot in 950 μl of PBS or physiologic solution.

The concentration and the size distribution of ZnO NCs, EVs and TNHs dispersed in 0.1-μm filtered different solutions (1:100 dilution factor) were measured by nanoparticle-tracking analysis (NTA) technique with a NanoSight NS300 (Malvern Panalytical) equipped with a λ = 505 nm laser beam and a NanoSight syringe pump. All the experiments were carried out by capturing three videos of 60 s, each with an infusion rate of 30 and a camera level between 11 and 15. Videos were analyzed with the NTA 3.2 software, setting the detection threshold at 5.

The just-prepared TNH samples were analyzed through fluorescence microscopy and quantified by a colocalization tool to evaluate the percentage of coupling between fluorescently labeled EVs and ZnO NCs. A 10-μl drop of the TNH solution was deposited on a glass microscope slide, covered with a cover glass slip (0.17-mm thick; VWR) and analyzed with a wide-field fluorescence-inverted microscope (Eclipse Ti-E, Nikon, Tokyo, Japan), equipped with a super bright wide-spectrum source (Shutter Lambda XL), a high-resolution camera (Zyla 4.2 Plus, 4098 × 3264 pixels, Andor Technology, Belfast, UK) using an immersion oil 60× objective (Apo 1.40, Nikon). The colocalization tool of NIS-Element software (NIS-Elements AR 4.5, Nikon) was used to evaluate the coupling percentages: after setting a threshold between 0.1 and 1 μm to disregard larger aggregates, the spots in the far-red (identifying the ZnO NCs) and green channels (corresponding to the EVs) were counted and an overlay of the two images was performed, counting only the spots in which the two fluorescences were colocalized. The percentage of colocalization was then calculated with respect to the ZnO channel, according to the following formula: % colocalized ZnO = (n° colocalized spots)/(Tot n° red spots).

Other colocalization percentages can be obtained on the basis of the EVs green channel or as an overall amount of the two channels, green and red, as detailed in Supplementary Table 2. Here, for brevity and validation, we only consider the % values obtained for the co-ZnO. Considering the ZnO NCs a nanodrug, it is necessary to optimize their incorporation into the EVs vesicles, therefore the %co-ZnO is monitored during the whole preparation step of the TNH and its maximization used as a measure of success.

### Live cell fluorescence microscopy

For *in vitro* experiments, the KB cells, treated with 15 μg/ml of TNHs and ZnO NCs and with the corresponding number ofEVs, were imaged after 24 h ofincubation at 37°C, 5% CO_2_. The cells were counted and seeded (3 × 10^4^ cells/well) in 4-well chamber slides (Nunc™ Lab-Tek™ II CC2™ Chamber Slide System, Thermo Fisher Scientific™, MA, USA). After 24 h of growth in standard conditions, the medium was replaced with 500 μl of treatment solution containing the particle for a further 24 h. To maintain healthy cells, in their physiological condition during the acquisition time, an incubator gas chamber (Okolab) equipped with CO_2_ sensors, temperature unit and an active humidity controller was used. In order to label cell membranes, 2.5 μl of wheat germ agglutinin conjugated with Alexa Fluor 488 dye (λ_Ex_ = 495 nm) was added to the cell samples and after 10 min of incubation at 37°C, 5% CO_2_, two washing steps were performed by using 500 μl of live-cell imaging solution (1×, Molecular Probes) at 37°C. ZnO NCs were used as labeled with Atto647 NHS ester dye, EVs were labeled in DiD and in DiO for sample treated with EVs and TNHs, respectively.

### Cell proliferation assay

KB cells were counted and 1.5 × 10^3^ cells/well were seeded onto a 96-well flat-bottom plastic culture plate (Corning, 96-well TC-treated microplates). After 24 h of growth at 5% CO_2_, 37°C, the culture medium was replaced with 100 μl/well of 5, 15, 25 and 50 μg/ml of ZnO NCs or TNHs and 5, 15, 25 μg/ml EVs treatment solutions. After 24 h treatments, the cell viability was assessed, 10 μl of the WST-1 (CELLPRO-RO Roche) was added to each well and after 2 h of incubation at standard conditions, the formazan absorbance was detected at 490 nm by the Multiskan Go microplate spectrophotometer (Thermo Fisher Scientific) using a 620-nm reference.

Cytotoxicity tests were carried out at least in triplicates and the viability values were normalized to control and expressed as mean ± standard error of the mean (SEM).

### Cell internalization assay

For measuring the TNHs, ZnO NCs or EVs internalization in KB cells, 3 × 10^4^ cells were cultured into a 24-well plate (Corning, 24-well TC-treated microplates) with 500 μl of complete cell culture medium for 24 h. Then, cell medium was replaced with freshly prepared solutions containing 5, 15, 25 μg/ml of TNHs (labeled with both DiO for EVs and Atto647 for the ZnO NCs) or, as control samples, with 5, 15, 25 μg/ml of ZnO NCs (labeled with Atto647) or with DiO-labeled EVs at the same concentration used for the preparation of 15 μg/ml of TNHs. Untreated KB cells were also prepared as reference. After 24 h, cells were washed with PBS, trypsinized, centrifuged at 130 × *g* for 5 min, and then resuspended in 500-μl PBS for the cytofluorimetric analysis. 1 × 10^4^ events were collected with a Guava Easycyte 6-2 L flow cytometer (Merck Millipore, MA, USA), with 0.59 μl/s flow rate, excluding cell debris. The analyses were performed by using the blue laser (λ_ex_ = 488 nm) to detect EVs and the red one (λ_ex_ = 642 nm) for ZnO NCs.

The positive events were characterized by a shift of Red-R fluorescence intensity (emission filter 661/15) for Atto647-ZnO NCs signal and a shift of Green-B fluorescence intensity (emission filter 525/30) for DiO-EVs signal. The percentages of positive events were compared with untreated cells, evaluated with Guava InCyte Software (Merck Millipore).

### Statistical analysis

The statistical comparison between the treatment groups was performed using one-way or two-way analysis of variance (ANOVA) with SIGMA Plot software. ***p < 0.001 and *p < 0.05 were considered significant. Independent experiments were performed three-times.

## Results

### ZnO NC characterization

**Figure 1:**
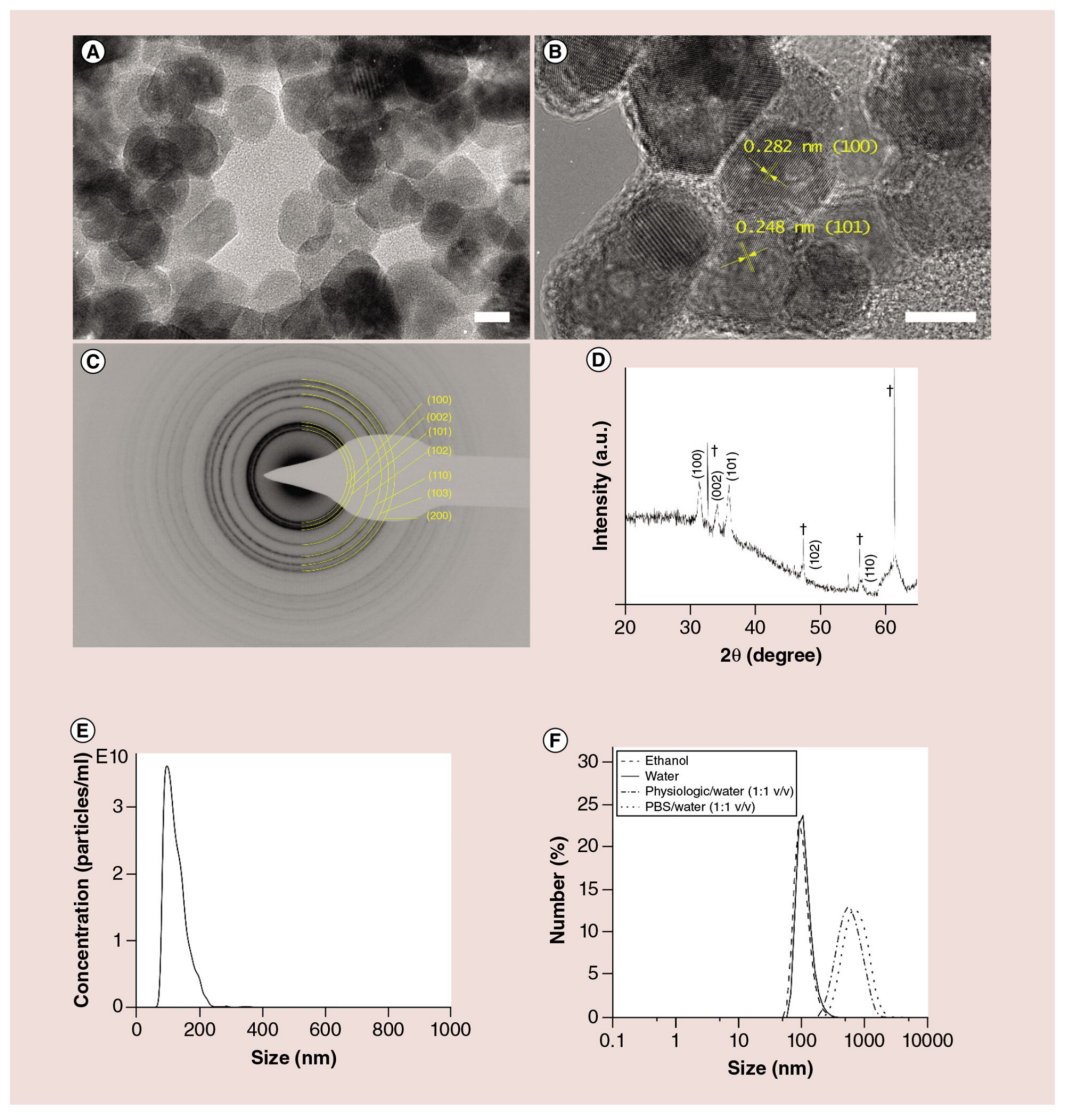
Zinc oxide nanocrystals characterization results. **(A)** Transmission electron microscopy and **(B)** high-resolution transmission electron microscopy images of the nanocrystals, scale bars are 10 nm. **(C)** Selected area electron diffraction pattern. **(D)** x-ray diffraction pattern. **(E)** Nanoparticle-tracking analysis of zinc oxide nanocrystals in double-distilled water. **(F)** Dynamic light scattering results of zinc oxide nanocrystals in different media (ethanol, double-distilled water, PBS and physiologic solution). ^†^Peaks of Si-wafer. PBS: Phosphate-buffered saline.

ZnO NCs were synthesized by a novel microwave-assisted solvothermal approach and functionalized with aminopropyl group as previously reported [30,35]. The amine groups are primarily used as efficient anchoring sites for dye labeling, in this case Atto647-NHS ester dye, to perform the fluorescence microscopy experiments. Therefore, the ZnO NCs used in this work are intended always as amine-functionalized ZnO NCs. The obtained tiny and highly crystalline NPs present a geometrical-rounded shape, with some hexagonal edges and an average diameter of 17 ± 0.2 nm, as visible by TEM in Figure 1A and B. They possess a single-crystalline structure corresponding to the wurtzite phase of ZnO (see the selected area electron diffraction [SAED] pattern in Figure 1C, the x-ray diffractogram in Figure 1D and [35] for further characterization details).

The size distributions of ZnO NCs obtained by both NTA and DLS characterizations are reported in Figure 1E and F, respectively. The NCs result well-dispersed in both ethanol (average hydrodynamic diameter: 91 nm) and in 100-nm filtered dd water (obtaining ZnO NCs average diameters of 103 and 99 nm from DLS and NTA, respectively). In Figure 1F, the DLS measurements of ZnO NCs dispersed in a 1:1 (v/v) solution of dd water and PBS or physiologic solution (0.9%w NaCl) report a rapid aggregation of ZnO NCs. In particular, the ZnO NCs reach an average hydrodynamic diameter of 550 and 720 nm in physiologic solution and PBS, respectively.

### EV characterization

**Figure 2:**
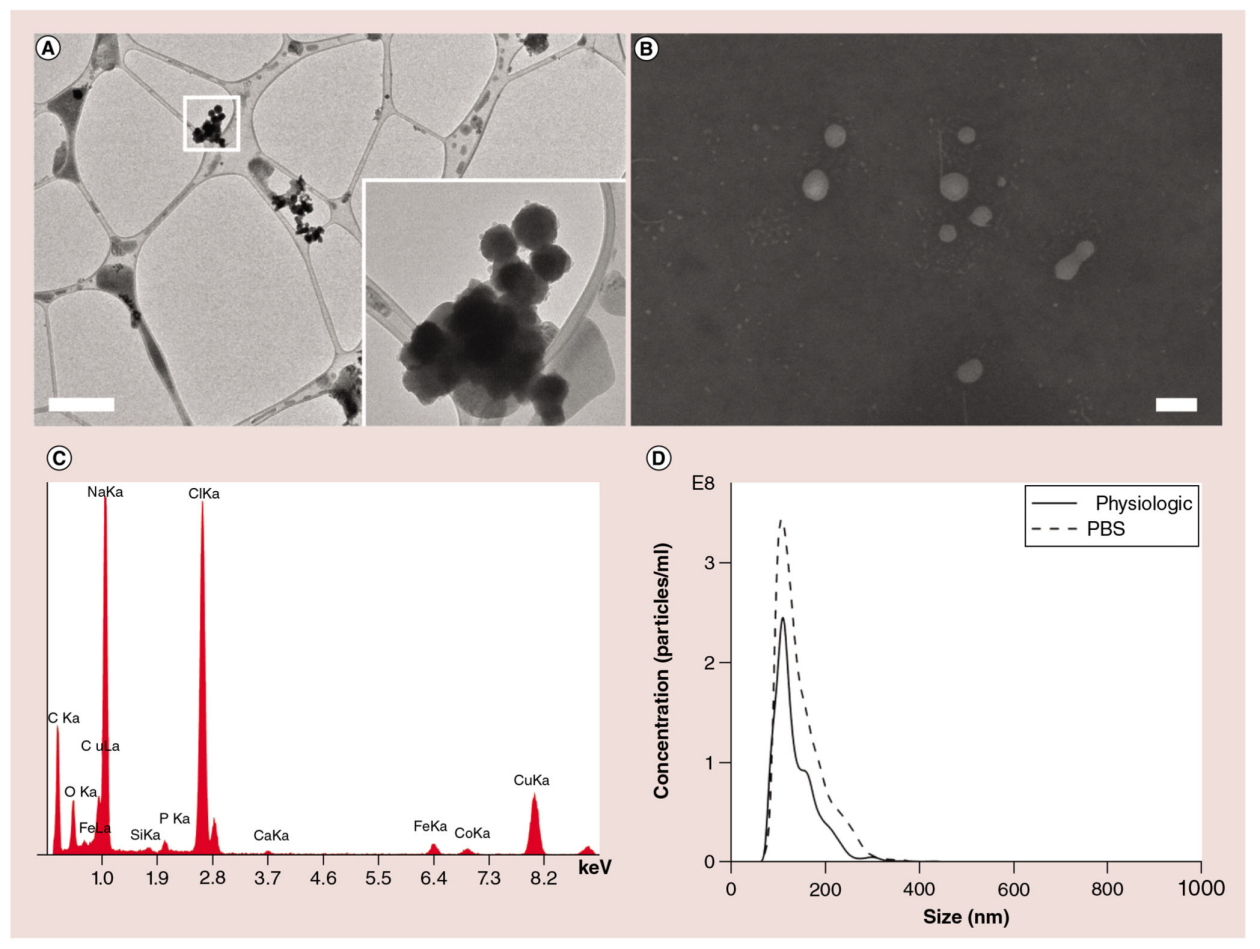
The extracellular vesicles extracted from KB cell culture. **(A)** Transmission electron microscopy image at 80 kV of freshly prepared extracellular vesicles and the magnification of a detail, scale bar is 1 μm. **(B)** Field emission scanning electron microscopy image of the ext., scale bar is 100 nm. **(C)** Energy dispersive spectroscopy analysis in the same region in **(A)**. **(D)** Nanoparticle-tracking analysis size distribution in PBS and physiologic solutions. PBS: Phosphate-buffered saline.

EVs extracted from cell culture supernatants (KB cell line, an oral epithelial carcinoma of the mouth) were also fully characterized, as reported in Figure 2. The images of EVs acquired with TEM at 80 kV (Figure 2A) and FESEM (Figure 2B) show round-shaped vesicles with a uniform diameter around 100 nm. TEM characterization shows that EVs are almost homogeneously distributed over the electron microscopy (EM)-grid with occasional overlap leading to some aggregated structures (Figure 2A). Energy dispersive spectroscopy (EDS) reports a representative analysis of the EV compositions (Figure 2C). Elements like carbon and oxygen are obviously expected, due to the lipid nature of the vesicles, as well as the high amount of sodium, chlorine and calcium derived from the physiologic solution used as dispersing media. The NTA reported in Figure 2D shows a uniform and narrow size distribution centered at 110 nm, in both physiologic and PBS solutions, very similar to the previous results reported by TEM and FESEM characterizations. Repeated NTA measurements (27 experiments) of EVs extracted from the same cell line reported similar size distributions with an average concentration of EV of 1.05 × 10^11^ particles/ml (ranging from a minimum of 1.5 × 10^10^ up to a maximum of 2 × 10^11^ particles/ml).

### TNH coupling: screening of processing parameters

**Figure 3:**
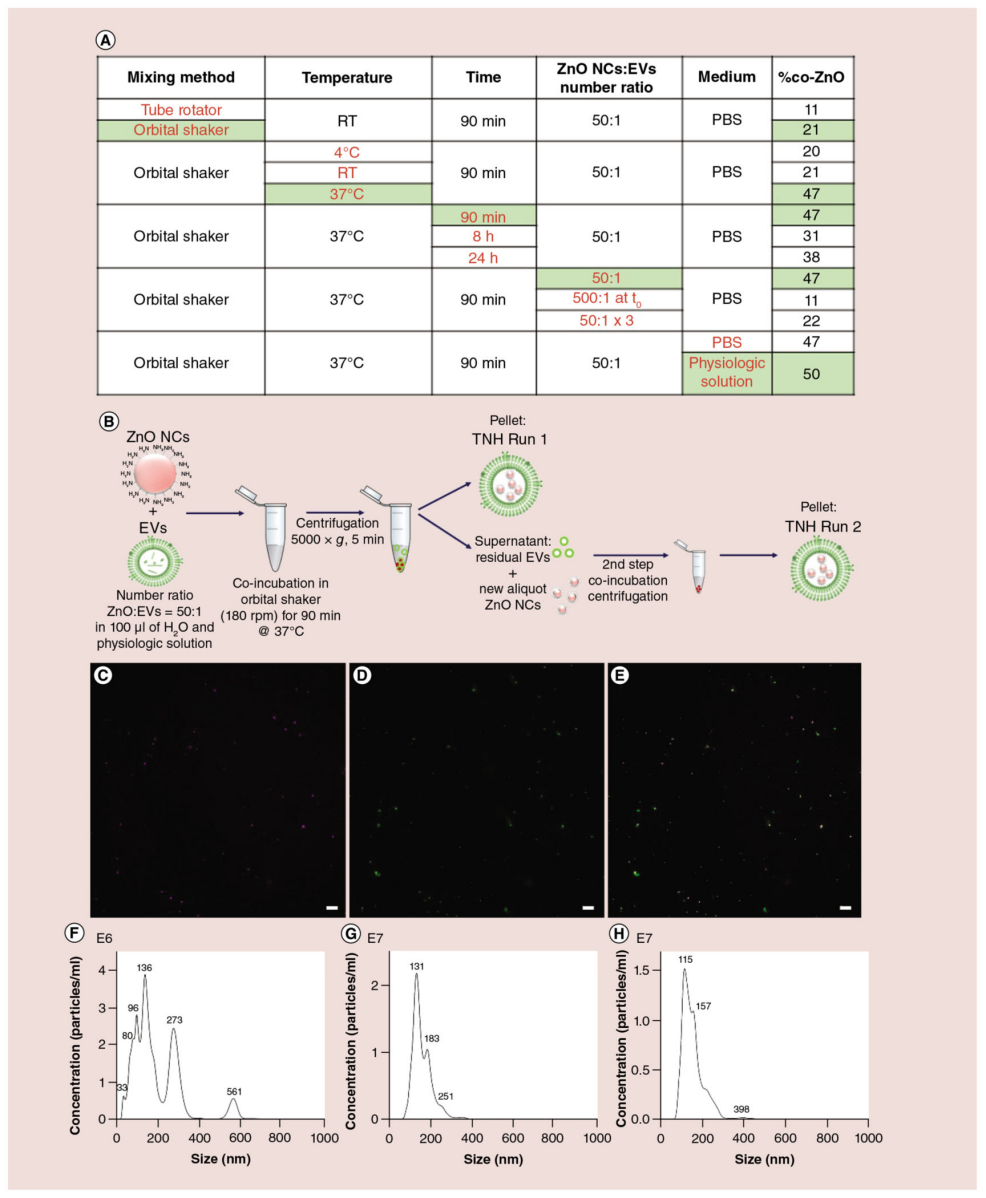
Coupling protocol and characterization of Trojan nano-horse. **(A)** Table of the %co-ZnO values obtained during the parameters’ screening process; the best evidenced choices are in green. **(B)** Graphical schematization of the optimized coupling process. **(C–E)** Examples of optimized TNH Run 1 fluorescence colocalization images. **(C)** In purple, ZnO NCs labeled with Atto647, **(D)** in green, extracellular vesicles labeled with 3,3’-dioctadecyloxacarbocyanine perchlorate; **(E)** the TNH as colocalized in the merged channel, scale bar is 10 μm. Nanoparticle-tracking analysis measurements of **(F)** ZnO NCs, **(G)** extracellular vesicles and **(H)** TNHs in 1:1 v/v double distilled water and physiologic solution. EV: Extracellular vesicle; PBS: Phosphate-buffered saline; RT: Room temperature; TNH: Trojan nano-horse; ZnO NC: Zinc oxide nanocrystal.

The coupling process between ZnO NCs and EVs was optimized investigating the influence of several operating parameters (i.e., the coupling temperature and time, the mixing method, the type of dispersing medium and the ratio between EVs and ZnO NCs) and quantifying the coupling efficiency through fluorescence microscopy. The results, summarized in Figure 3A, are reported as percentage of colocalization with respect to the ZnO-labeled red channel as explained in detail in the Materials & methods section. The %co-ZnO values are an average of the two runs performed during the coupling process (as sketched in Figure 3B). More colocalization percentages (%co-EVs and %TNH) are reported in Supplementary Table 2.

First, two mixing methods were tested, an orbital shaker set at 180 rpm and a tube-rotator with a fixed speed of 20 min^-1^. The very low coupling percentages obtained with the tube rotator (see Figure 3A) are attributed to the intrinsic continuous turning upside-down of the tube containing the solution, causing the spread of EVs and the NCs on the walls of the tube and reducing the probability of collisions between them and thus the coupling. Higher percentages (highlighted in green in Figure 3A) identify the orbital shaker as the most suitable mixing method, used in all subsequent experiments.

Another significant operating parameter tested in this screening process was the temperature of the coupling, varied between 4°C, RT and 37°C. As highlighted in green in Figure 3A, the best coupling efficiency occurs at the physiologic temperature of 37°C.

Lengthening the duration of the coupling procedure to 8 or 24 h did not remarkably increase the coupling percentages and the best percentages were obtained for runs of 90 min each.

Furthermore, Figure 3A reports the effect of the number ratio of ZnO NCs with respect to the EVs. Regarding this parameter, two sets of data have to be defined: ZnO NCs added at the beginning of the process (t = 0 h) in a different number ratio with respect to the EVs (50:1 and 500:1); ZnO NCs added in 50:1 ratio consecutively (at time steps t = 0, 1, 2 h, where t = 0 h is the beginning of the process), for a final ratio of 150:1 with respect to the EVs.

In the first experiment, the ratio 50:1 of ZnO:EVs is preferred in terms of coupling percentages achieved with respect to the 500:1 one. Also the second experiment highlights the 50:1 ZnO:EVs at the beginning of each run as the preferable ZnO:EVs ratio, since no appreciable improvement was observed in the case of three consecutive additions of ZnO NCs within the same run.

Finally, tests were carried out to understand which dispersing medium was the most suitable for an optimal coupling, analyzing two buffers (PBS and physiologic solution) diluted at a ratio 1:1 in volume with dd water. As shown in the last line of the table in Figure 3A, the higher coupling efficiency was obtained with the experiments performed in physiologic solution. To analyze the influence of the dispersing media on the surface properties of the TNH components, ζ-potential measurements were performed. The obtained values show that EVs always have a negative potential (-9.53 mV), whereas the ZnO NCs shift from a negative value in PBS/H_2_O (-15.5 mV) to a positive one in physiologic solution/H_2_O (+12.3 mV).

### ZnO & EV optimal coupling conditions & TNH characterization

From the collected colocalization percentages, the optimal conditions to maximize the coupling efficiency between ZnO NCs and EVs were defined as follows: two runs of coupling combining a 50:1 number ratio of ZnO:EVs at each run. The total volume used was 100 μl for each run composed of a solution 1:1 v/v of dd water and physiologic solution (0.9% NaCl). Each run was then carried out at 37°C under orbital shaking (180 rpm) for 90 min. The optimal coupling process is sketched in Figure 3B and the fluorescence images of the red, green and merged channels of TNH Run 1 obtained are reported as an example (Figure 3C–E). After Run 1, the highest colocalization of 59%co-ZnO was achieved, whereas after Run 2, 22%co-ZnO was obtained, owing to the reduced amount of residual EVs in the solution. As an average between the two runs, we can estimate a colocalization percentage of 40%co-ZnO. More details of the optimal coupling process are reported in Supplementary Table 3 and Supplementary Figure 1.

**Figure 4:**
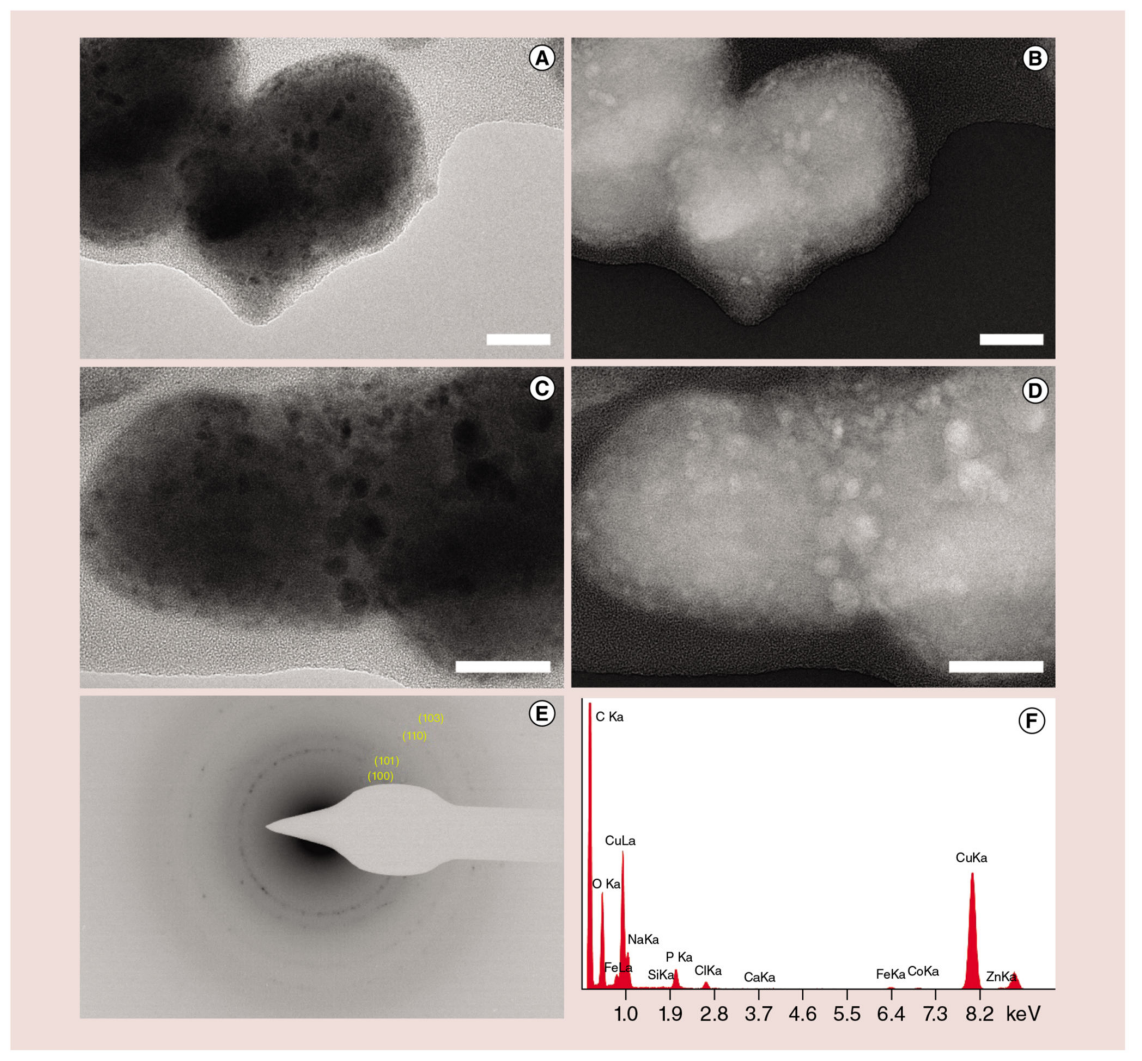
Electron microscopy images of the Trojan nano-horse construct. Trojan nano-horse imaged by transmission electron microscopy at 80 kV, freshly prepared without further fixing or staining process **(A–D)**, scale bars are 30 nm. **(B)** and **(D)** are the same pictures as **(A)** and **(C)**, but with inverted colors to improve the zinc oxide nanocrystal visibility. **(E)** Selected area electron diffraction pattern and **(F)** energy dispersive spectroscopy.

The TNH samples obtained with the optimized protocol were further characterized by TEM, NTA and ζ-potential measurements. The morphological characterization was carried out by TEM at 80 kV on freshly prepared TNHs without any further fixing or staining (Figure 4A–D & Supplementary Figure 2).

The images show round-shaped EVs, with some overlaps between them, incorporating (or immobilizing at their surface) dense and crystalline structures of around 15 nm, corresponding to ZnO NCs. To better visualize the tiny NCs embedded into the EVs, we provide the same TEM images with inverted colors (Figure 4–D). In a further experiment, we imaged the freshly prepared TNH at 200 kV (Supplementary Figure 2), observing the progressive melting of the organic phase and leaving the ZnO NCs arranged in a circular fashion, recalling the EV which they were embedded in.

The SAED pattern (Figure 4E) confirms the presence of crystalline structures with the d-spacing typical of ZnO nanomaterials (see also Figure 1C for comparison with the SAED pattern of pristine ZnO NCs). The EDS analysis in Figure 4F shows the clear presence of Zn element in the prepared TNH sample, together with the other elements detected in the pristine EVs. As a comparison, the pristine EVs do not report any signal of Zn in their respective EDS spectrum (Figure 2C). All these results, if compared with the bare ZnO NCs (Figure 1) and the pristine EVs (Figure 2), together with the fluorescence colocalization microscopy images (Figure 3C–E), confirm the effective loading of ZnO NCs within the EVs.

Furthermore, the final ζ-potential value of the TNH in physiologic solution/H_2_O (1:1 v/v) is -13.2 mV. This value is close to the one obtained for the pure EVs (-9.5 mV, see above) and far from the positive value obtained for the ZnO NCs in the same solution (+12.3 mV).

In order to evaluate the eventual improvement of the colloidal biostability of ZnO NCs provided by the EVs shielding, NTA measurements were performed. The recorded size distributions of TNHs, as well as of pristine ZnO NCs and EVs in 1:1 dd water: physiologic solution are reported in Figure 3F–H. By comparing the size distribution of ZnO NCs (Figure 3F) in 1:1 dd water:physiologic solution with the one in Figure 1E, reporting the same NCs dispersed in pure dd water, it clearly shows that the ZnO NCs tend to aggregate in presence of physiologic solution, with the appearance of peaks at higher size values (273 and 561 nm). Strikingly, the TNH sample (Figure 3H) results are highly dispersed in the same 1:1 dd water and physiologic solution, with a size distribution centered at 115 nm and two minor peaks at 157 and 398 nm. The TNH distribution clearly resembles the one obtained for the uncoupled EVs (Figure 3G) in the same medium.

### TNH, ZnO NC & EV cytotoxicity & cellular internalization in KB cells

**Figure 5:**
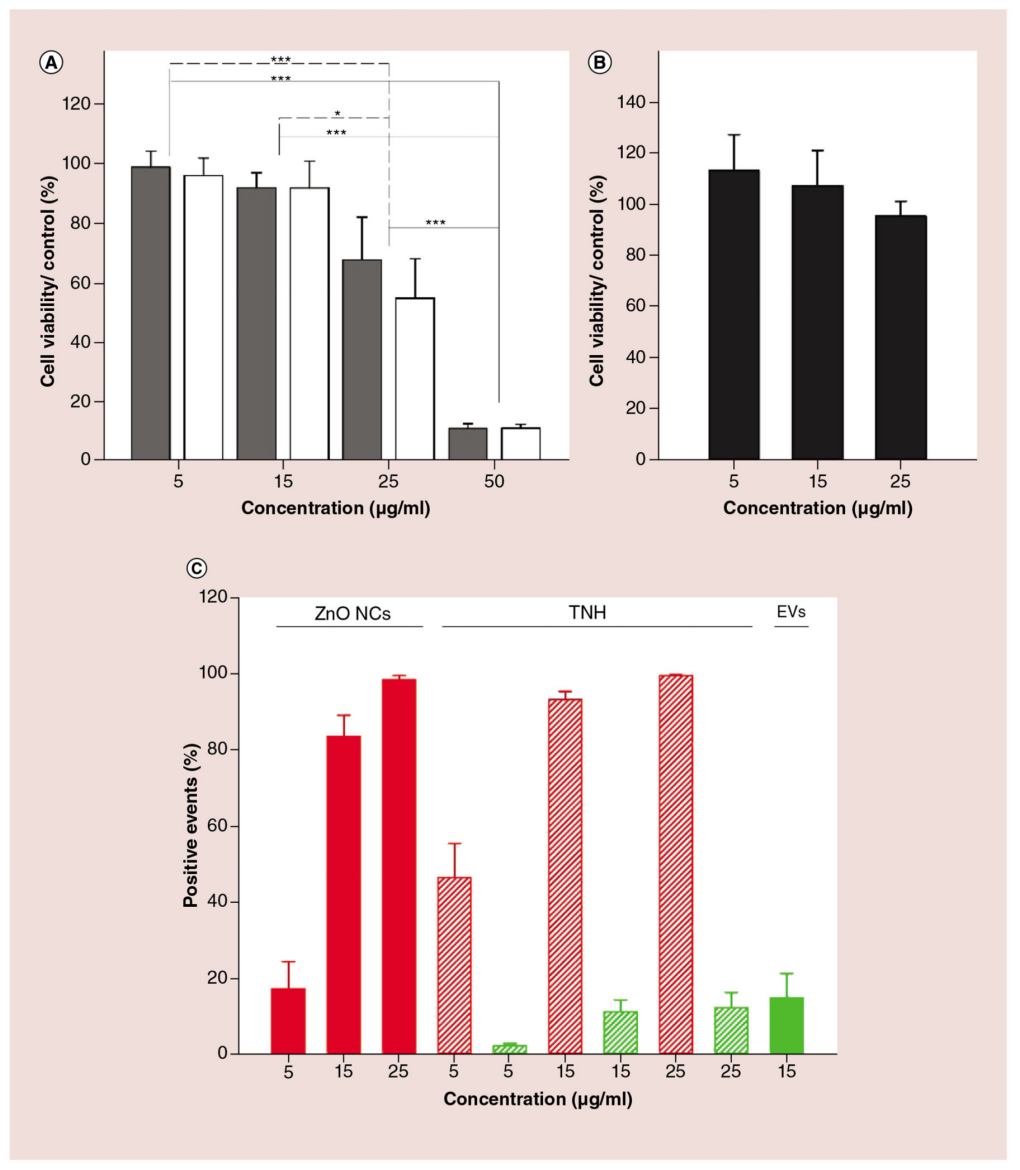
Trojan nano-horse, pristine Zinc oxide nanocrystals and extracellular vesicles cytotoxicity and cell internalization in KB cells. Cell viability after 24 h treatment with different concentration of **(A)** ZnO NCs (gray bars), TNHs (white bars) and **(B)** EVs (black bars) assessed with the WST-1 assay. **(C)** Internalization flow cytometry-based assay results. The histograms represent the percentages of fluorescence positive events normalized on the untreated cells. Red bars represent the Red-R percentages of positive events for cells incubated with different concentrations of ZnO NCs. Red and green dashed bars represent the Red-R and Green-B-positive events, respectively, for cells incubated with the different concentrations of TNHs. The EVs histogram (green) shows the Green-B percentage of positive events after treatment with a 15 μg/ml solution of EVs. Number of assays at least two. Comparisons were performed using the two-way analysis of variance for **(A)** and **(C)** and the one-way analysis of variance for **(B)**. *p < 0.05, ***p < 0.001 and values represent the mean ± standard error bars. EV: Extracellular vesicle; TNH: Trojan nano-horse; ZnO NC: Zinc oxide nanocrystal.

The viability of KB cells was evaluated by WST-1 assay following incubation with various concentrations of TNHs, ZnO NCs and EVs for 24 h. In Figure 5A, cell viability values are reported as percentage growth relative to the untreated control. ZnO NCs and TNHs significantly (p ≤ 0.001) inhibited cell viability when tested at 25 and 50 μg/ml. Given the results of cytotoxicity tests on ZnO NCs and TNHs, the same assay was performed on isolated EVs to assess their full biocompatibility. The most toxic concentration (corresponding to 50 μg/ml of ZnO NCs), ineligible for theranostic applications of the TNH, was excluded from the EV tests. One-way ANOVA on data shown in Figure 5B confirmed that treatment with pristine EVs did not affect the cell viability (p = 0.667).

To further deepen the understanding of the TNH mechanism of action, we also explored the internalization rate of the various nanoconstructs at different concentrations. Flow cytometry (Figure 5C) allowed single-cell analysis of TNHs, ZnO NCs and EVs internalization in KB cells after 24 h incubation. It was observed that higher percentages of Red-R-positive events (related to the fluorescence of the dye bound to ZnO) were obtained in cells incubated with a higher concentration of both TNHs and ZnO NCs (Figure 5 C, red dashed and red bars, respectively). These results are evidence that TNHs are more efficiently internalized in KB cells (p < 0.05) allowing the entrance of larger amount of ZnO NCs if compared with the pristine ZnO NCs without EVs coating, especially for low dosages. Furthermore, the two-way ANOVA confirmed a significant (p < 0.001) dose-dependent variation in the internalization of ZnO NCs and TNHs that experimentally reached a plateau at 15 μg/ml. It should be noted that ANOVA analysis of Green-B-positive events, related to the fluorescent dye DiO labeling the EV lipids, did not show a dose-dependent effect comparing the internalization for 5, 15 and 25 μg/ml TNH or 15 μg/ml EVs treatments.

**Figure 6:**
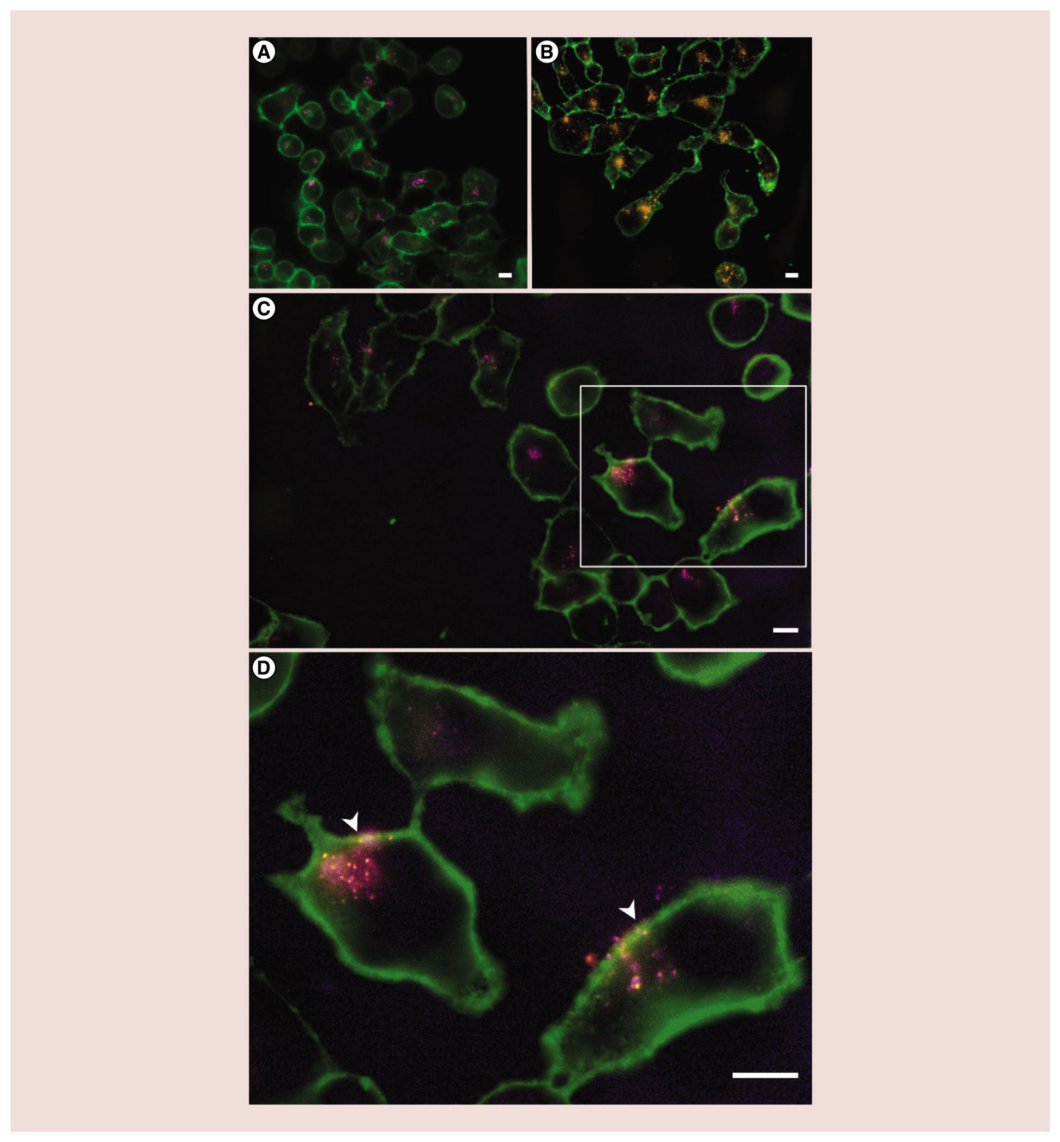
Cellular uptake of nanoparticles by KB cells. Fluorescence microscopy z-stack images of the internalized **(A)** zinc oxide nanocrystals, **(B)** extracellular vesicles and **(C)** Trojan nano-horses. The cell membranes are depicted in green, extracellular vesicles in orange and zinc oxide nanocrystals in purple. **(D)** Represents the enlargement of the area in the white rectangle to highlight the colocalization between zinc oxide and extracellular vesicles; scale bars are 10 μm.

Fluorescence microscopy actually confirmed the reproducible internalization of TNHs as well as of pristine ZnO NCs and EVs in the KB cell line (Figure 6). It should be also noted that the percentage of positive events regarding the DiO green fluorescence obtained with the cytofluorimeter was lower compared with those observed in fluorescence microscopy. This was probably due to the slight green autofluorescence found in KB cells (untreated cells possess have a high-background signal in the Green-B channel) and the shift in fluorescence intensity in cells internalizing EVs was less pronounced than those recorded in Red-R channel of the cytofluorimeter. In our case, the fluorescence microscope, equipped with a couple of filters able to cut out typical cellular cytoplasmatic autofluorescence, allowed a better differentiation of DiO green signal from the cell background if compared with flow cytometry.

To further gain insights into the different uptake behavior and investigate the internalization mechanism of the TNH inside KB cancer cells, in comparison with the pristine ZnO NCs and EVs as reference nanomaterials, the cells were incubated with 15 μg/ml of TNHs and ZnO NCs and with the corresponding amount of EVs (see the Materials & methods section) and monitored by fluorescence microscopy after 24 h of incubation. The fluorescence microscope imaging, selected by z-stacking, showed a clear uptake of both ZnO NCs (Figure 6A) and EVs (Figure 6B) at the end of the incubation time. When present, the aggregates clearly contrasted from the cells as bright dots. Both ZnO NCs or EVs internalized by KB cells preferentially accumulated in a cytoplasmatic perinuclear area, separately or as small aggregates. Considering different regions of interest for each sample, the KB cells uptaking the EVs showed higher uptake rate than those treated with ZnO NCs.

The TNHs (Figure 6C) revealed a similar internalization behavior like those presented by the two reference components, in other words, ZnO NCs and EVs. KB cells showed the selective internalization of few structures, but very well recognizable in the cytosolic space as purple and green spots, as well as partially colocalized structures (yellow spots). The magnification reported in Figure 6D highlights a partial coupling between the ZnO NCs and the EVs: several purple spots, representing the ZnO NCs labeling, in the cytoplasmatic space poorly colocalize with the EVs fluorescence. The white arrows in Figure 6D show a high colocalization between the cell membrane and the TNHs, evidencing the entering of these nanoconstructs inside the cells.

## Discussion

ZnO NCs were efficiently prepared by a reproducible wet-chemical synthesis assisted by microwaves. Their functionalization with amino-propyl groups does not induce morphological changes but actually provides a positively charged surface on the NCs, useful not only to improve the colloidal stability of the NCs in solution but also to elicit an electrostatic interaction with the negatively charged EVs surface [10,35,36], as better described below.

To increase the ZnO NCs biocompatibility and biostability in biological media, a method for an efficient internalization in EVs was investigated for further theranostic applications.

Starting from the literature review concerning the encapsulation of drugs in EVs [37], as well as recent coupling experiments between exosomes and gold [23,24] or metal organic framework [25] NPs intended for imaging or drug delivery purposes, we tuned various parameters to efficiently load the EVs and extracted from KB cell culture supernatants with ZnO NCs. The optimized coupling protocol exploits a combination of various mechanisms, including thermodynamic, kinetic and electrostatic ones, specifically linked to the EVs nature and to the NCs morphology, size, electrostatic charge, crystallinity and chemical structure.

The temperature mainly affects the fluidity of the phospholipidic membrane of the EVs, which must be fluid enough to envelop the ZnO NCs, allowing the entrance of such solid NPs. Therefore, the increased rigidity of the phospholipidic membranes of EVs at 4°C, compared with higher temperatures, does not allow ZnO NCs to efficiently penetrate them, as demonstrated by the low colocalization percentages obtained. In contrast, at higher temperatures (i.e., RT and 37°C), better coupling percentages are obtained suggesting that the incorporation of ZnO NCs into the phospholipidic membrane involves a thermodynamic process.

Concerning the duration of the coupling procedure, several hours of coupling, 8 or 24 h, did not remarkably increase the coupling percentages, suggesting that the kinetics of coupling is high enough to perform it in a relatively short time period. In both cases, the coupling percentages after 8 and 24 h were lower than after 90 min of coincubation, probably due to an aggregation of the ZnO NCs in the suspension medium. Furthermore, for both 8 and 24 h coupling times, the second run was not performed, due to the already prolonged time of processing of the first run. Therefore, short coupling cycles are preferred to obtain TNHs and avoid possible EVs deterioration or contamination before their *in vitro* or *in vivo* applications.

The tendency of ZnO NCs to aggregate in the used solutions is also related to ZnO NC concentration and in particular is enhanced by its increase. Therefore, better experimental results were obtained by performing multiple runs, adding the ZnO NCs each time in a ratio of 50:1, than a single addition of a larger amount of NCs. Also the consecutive additions of multiple aliquot of ZnO NCs within the same run did not remarkably improve the colocalization percentages compared to the usual addition of 50:1 ZnO:EVs at the beginning of each run, highlighting a clear limit of NCs loading within the EVs.

Finally, we hypothesize that the influence of dispersing medium is mainly attributable to electrostatic surface charge mechanisms. In fact, the composition of the media can affect both the colloidal stability of the ZnO NCs and the ζ-potential values of the NCs in the above-mentioned solutions. As depicted in Figure 1F, the DLS size distributions of the ZnO NCs show a slightly lower extent of aggregation in physiologic solution than in PBS. Most importantly, the ζ-potential values clearly reported that EVs always show a negative potential, whereas the ZnO NCs shift from a negative value in PBS/H_2_O to a positive one in physiologic solution/H_2_O. This finding suggests that the electrostatic interactions between EVs and ZnO NCs may also play an important role. In fact, the positive potential of the ZnO NCs in physiologic solution can promote an electrostatic interaction toward the EVs membrane, enhancing the coupling’s efficiency.

The TNHs obtained with the optimized procedure, defined on the basis of the above considerations, were further characterized confirming both the success of the developed coupling procedure and the stabilization effect of the EVs shielding on the ZnO NCs colloidal behavior. From TEM images, in fact, the NCs seem to be almost homogeneously distributed throughout the EVs volume or immobilized at their surface, while the NTA measurements, performed on both TNHs and ZnO NCs, show that the EVs shielding efficiently prevents the aggregation of pristine NCs in physiological solution. Also, the negative ζ-potential value obtained for the TNH in physiologic solution/H_2_O (1:1 v/v), closer to the one obtained for the pure EVs and far from the positive value of the ZnO NCs in the same solution, constitutes evidence of the success of the coupling procedure. The slight variation of the ζ-potential value between TNH and pristine EVs, can be attributed to some rearrangement of the polar heads of the phospholipids of EV membranes, caused by the ZnO NCs insertion into the vesicle. This effect was also reported in the literature concerning EVs loaded with drugs or photosensitive components [37,38].

Through *in vitro* studies, we have demonstrated efficient intracellular delivery to cancer cells. Therefore, the TNHs showed the same cytotoxic capability of the pristine ZnO NCs, demonstrating their potential as therapeutic nanotools, since the coupling with EVs did not affect ZnO NCs intrinsic cytotoxicity.

Actually, from fluorescence microscopy experiments, it seems that the TNHs are able to cross the plasma membrane through very close contact with it. It can thus be assumed that part of the phospholipidic components remain at the cell membrane or destabilizes inside the cell, leaving the ZnO NCs as ‘naked’ pristine nanomaterial in the cytoplasmatic space. It is therefore clear that the ‘naked’ ZnO NCs can then efficiently elicit a cytotoxic response in the cancer cell, as the reference material in the control experiments can.

These results strongly support the observed levels of cytotoxicity of TNH; similar to those of the pristine ZnO NCs. Hence, TNHs cytotoxic activity can be attributed to the ZnO core, as described in the previous literature on ZnO NCs [10,26,35]. ZnO cytotoxicity can be related to three main events: the ZnO intracellular dissolution and release of effective amount of Zn^2+^ ions, the production of ROS and the direct damage of membrane structures.

The comparable cytotoxic effect between the pristine ZnO NCs and the TNHs after 24 h suggests that, in the TNH nanoconstruct, the membrane of the EVs could not remain stable around the ZnO NCs once entered the cell. As reported in the literature [39,40], the main fate of EVs after internalization is the degradation and the recycling for cell physiological functions.

In this scenario, we could hypothesize that the lipid coating of our TNHs does not last long enough to prevent ZnO NCs dissolution and interaction with membrane components, producing cytotoxic effects. However, the successful incorporation of the NCs inside the EVs, creating a TNH, can efficiently promote their colloidal stability in biological fluids and consequently high internalization’s level into cancer cells.

## Conclusion

To conclude, here we demonstrate that the ZnO NCs can be efficiently incorporated and biostabilized within the lipidic bilayer of cell-derived EVs. Their inclusion into the EVs allows their stabilization toward degradation and aggregation in biological media, overcoming a common problem faced when formulation of NPs or drugs are used. Our findings demonstrate that the cytotoxic nanodrug, ZnO NCs, can be delivered to cancer cells in a reproducible, rapid and efficient way. During and/or after cellular uptake processes, partial EV membrane destabilization takes placed leaving the pristine ZnO NCs in the cytoplasm and thus able to elicit a cytotoxic response in the cancer cell. Further tracking studies of the nanodrug delivery from the EV carrier are required for a full understanding of the internalization mechanism in eukaryotic cell lines.

Strikingly, the TNH nanoconstruct is therefore ideal for the inclusion of many and even different NCs with biomodulatory, cytotoxic and antiproliferative capabilities inside autologous or heterologous EVs. The reengineering of EVs thus allows to create potentially low immunogenic, highly stable, hemocompatible nanoplatforms, with customizable targeting ability able to carry cytotoxic species, in this case nanocrystalline materials, intended as nanodrugs.

## Future perspective

To further exploit both the therapeutic and diagnostic potential of the proposed TNHs, we suggest the following improvements. A better purification procedure, based on immune-selection and isolation, could be utilized to obtain a higher coupling percentage and remove all the uncoupled ZnO NCs. However, a lower yield of the final TNH nanoconstructs should be then taken into account.

The modularity of the proposed TNH approach allows incorporation of inorganic materials, or even anticancer drugs into EVs, together with the ZnO nanodrug proposed here. Furthermore, including semiconductor nanocrystalline material, such as ZnO, allows exploitation of its optical light emission properties as a molecular luminescent probe for cell imaging, as already demonstrated [35,41], and upon selective targeting, for diagnosis purposes.

It is also likely to improve the selectivity of TNH targeting capability toward the desired cells or tissues. Although relying on the natural homing function of the EVs, the integration of targeting ligands (antibodies or peptides) can lead to a faster and more specific internalization and delivery of the bioactive cargo, that is – the ZnO nanodrug or other drug molecules, growth factors or nucleic acids into the cell.

In general, it is clear that the nanomedicine approach will broadly benefit from improving the colloidal dispensability of artificial nanoconstructs [5], like the TNH proposed here, and their targeting capabilities, affecting the final biodistribution *in vivo*. All these parameters are fundamental factors to increase the chances of these nanoconstructs to better perform in terms of therapeutic and diagnostic abilities, avoiding any potential side effects.

## Supplementary Material

Supplementary Material Supplementary data
To view the supplementary data that accompany this paper please visit the journal website at: www.futuremedicine.com/doi/suppl/10.2217/nnm-2019-0231

## Summary points

- Pristine zinc oxide nanocrystals (ZnO NCs) demonstrate a high and preferable toxicity toward cancer cells, but they are not stable in biological fluids, showing a high aggregation rate.
- The coupling efficiency of ZnO NCs with extracellular vesicles (EVs) can be improved by varying kinetic, thermodynamic and electrostatic parameters.
- EV shielding promotes the colloidal stability of pristine ZnO NCs in biological fluids.
- The coupling with EVs does not affect the intrinsic cytotoxicity of ZnO NCs.
- EV coating ensures a high internalization level inside cancer cells in a concentration related way.
- Trojan nano-horses can interact with cells by crossing their membranes and they preferentially accumulate in the cytoplasmic perinuclear area.
