# Supplementary material for: ZnO nanocrystals shuttled by extracellular vesicles as effective Trojan nano-horses against cancer cells
Source: Nanomedicine (Lond). Author manuscript; Available in PMC 2021 Apr 7. (PMC7610546; doi:10.2217/nnm-2019-0231)
Supplement: Supplementary Material — Supplementary data To view the supplementary data that accompany this paper please visit the journal website at: www.futuremedicine.com/doi/suppl/10.2217/nnm-2019-0231 [file EMS121176-supplement-Supplementary_Material.zip › suppl_data/suppl_data.docx]

**Supplementary Information**

ZnO nanocrystals shuttled by extracellular vesicles as effective Trojan nano-horses against cancer cells.

**Table S1**. **TNH preparation for biological assays.** Amounts of ZnO NCs and EVs, as well as solutions and related volumes used for the TNH preparation at various concentration for the biological essays with KB cancer cells.

| Treatment | Run 1  (90’ @ 37°C, 180 rpm) |  | Centrifugation  (5’000 g, 5min) | Run 2  (90’ @ 37°C, 180 rpm) |
| --- | --- | --- | --- | --- |
| 5 µg/ml | 2.5 µg ZnO NCs in 25 µl H_2_O  + 31.5 µl EVs in physiologic solution (conc: 1.9·10^11^ part/ml) | +283.5 µl physiologic solution | -Pellet + 635 µl EMEM | Supernatant  + 2.5 µg ZnO NCs in  25 µl H_2_O |
|  |  |  | -Supernatant: at Run 2 |  |
| 15 µg/ml | 7.5 µg ZnO NCs in 25 µl H_2_O  + 94 µl EVs in physiologic solution (conc: 1.9·10^11^ part/ml) | +221 µl physiologic solution | -Pellet + 635 µl EMEM | Supernatant  + 7.5 µg ZnO NCs in  25 µl H_2_O |
|  |  |  | -Supernatant: at Run 2 |  |
| 25 µg/ml | 12.5 µg ZnO NCs in 25 µl H_2_O  + 157.5 µl EVs in physiologic solution (conc: 1.9·10^11^ part/ml) | +157.5 µl physiologic solution | -Pellet + 635 µl EMEM | Supernatant  + 12.5 µg ZnO NCs in 25 µl H_2_O |
|  |  |  | -Supernatant: at Run 2 |  |
| 50 µg/ml | 25 µg ZnO NCs in 25 µl H_2_O  + 315 µl EVs in physiologic solution (conc: 1.9·10^11^ part/ml) | - | -Pellet + 635 µl EMEM | Supernatant  + 25 µg ZnO NCs in  25 µl H_2_O |
|  |  |  | -Supernatant: at Run 2 |  |

**Table S2.** **TNH colocalization percentages.** Colocalization percentages of the two averaged runs of coupling between ZnO NCs and EVs, evaluated after varying different processing parameters: mixing method, temperature, time, medium used, and ZnO NCs:EVs ratio in number of particles (evaluated from NTA measurements). The rows showing the best colocalization percentages in at least one channel with respect to the considered parameter are highlighted in green.

| **Varied parameters** | | **Colocalization percentages** | | |
| --- | --- | --- | --- | --- |
|  |  | **%co-ZnO NCs** | **%co-EVs** | **%TNH** |
| **Mixing Method** | Tube rotator | 11 | 22 | 6 |
|  | Orbital shaker | 21 | 28 | 13 |
| **Temperature** | 4°C | 20 | 27 | 14 |
|  | RT | 21 | 28 | 13 |
|  | 37°C | 47 | 25 | 18 |
| **Time** | 90’ | 47 | 25 | 18 |
|  | 8h | 31 | 16 | 12 |
|  | 24h | 38 | 8 | 7 |
| **ZnO NCs:EVs number ratio** | 50:1 | 47 | 25 | 18 |
|  | 500:1 at t0 | 11 | 18 | 7 |
|  | 50:1 x 3 | 22 | 24 | 13 |
| **Medium** | PBS | 47 | 25 | 18 |
|  | Physiologic solution | 50 | 25 | 21 |

The colocalization percentages reported in table S1 are calculated through colocalization tool of NIS-element software (NIS-Elements AR 4.5, Nikon), setting a threshold between 0.1 and 1 µm to disregard larger aggregates, according to the following formulas:

$$\% colocalized ZnO= \frac{n^{\circ} colocalized spots}{Tot n^{\circ} red spots}$$

$$\% colocalized EVs = \frac{n^{\circ} colocalized spots}{Tot n^{\circ} green spots}$$

$$\% overall TNH= \frac{n^{\circ} colocalized spots}{tot n^{\circ} red spots + tot n^{\circ} green spots - n^{\circ} colocalized spots}$$

Basically, the percentages are reported for each single fluorescence channel, i.e. the %co-ZnO and %co-EVs are the percentages of colocalized spots with respect to the total amount of distinguishable red-labelled ZnO NCs and green-labelled EVs spots respectively. Furthermore, the overall colocalization percentage, i.e. %TNH, that is the amount of colocalized spots with respect to all fluorescent events, are also calculated.


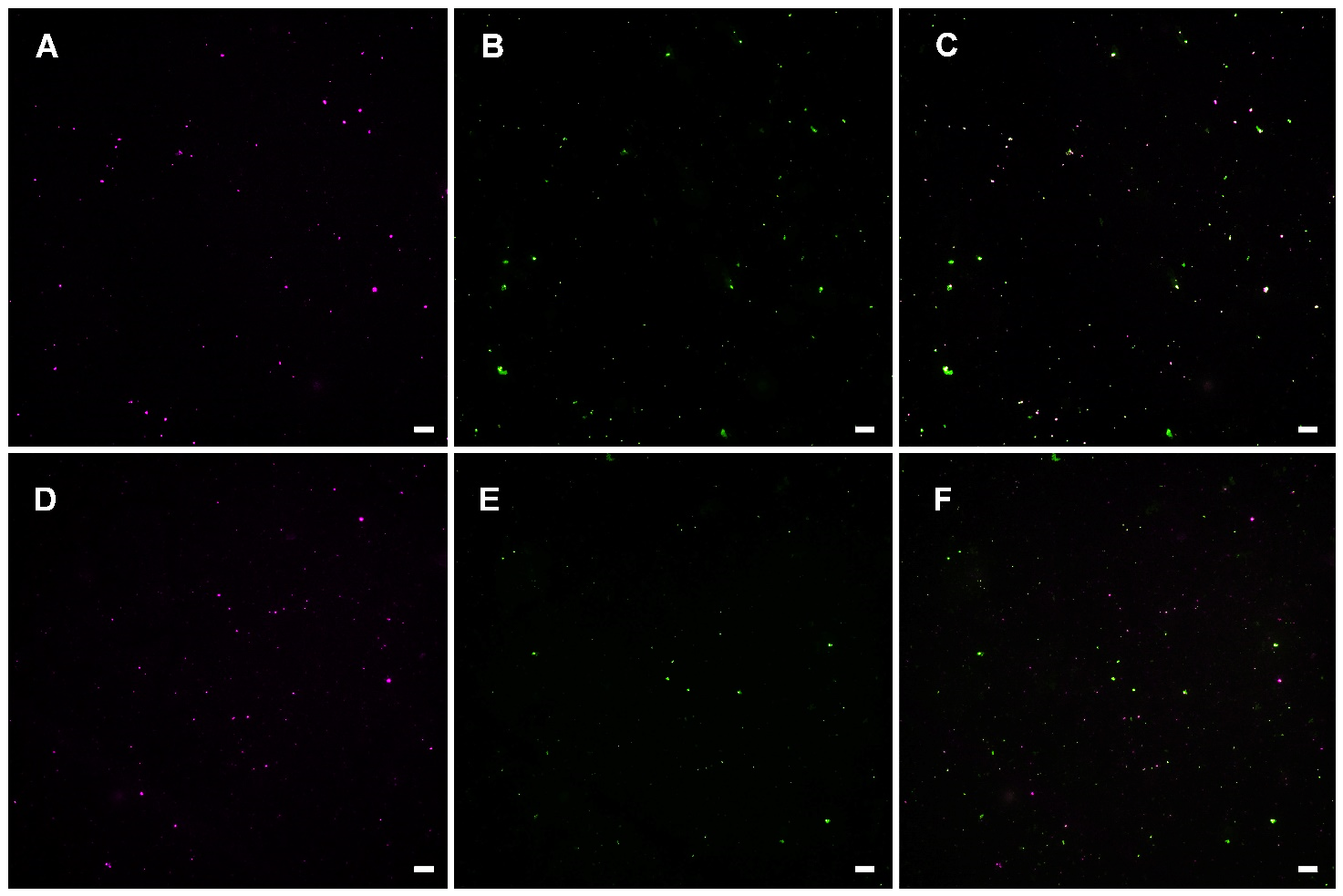


**Fig. S1.** **Fluorescence images of the two runs of coupling of TNH nanoconstructs.** Fluorescence colocalization images of ZnO NCs labelled with Atto647, EVs labelled with DiO and TNH as colocalized spots in the merged channel in TNH run 1 (**A**, **B** and **C** respectively) and TNH run 2 (**D**, **E** and **F**) from the optimized coupling procedure.

**Table S3.** **Optimized colocalization percentages.** Colocalization percentages results obtained for the optimized TNH coupling process, referred to the averaged results from tens of fluorescence co-localization images, as exemplified in Figure S1.

|  | **% co-ZnO NCs** | **% co-EVs** | **% TNH** |
| --- | --- | --- | --- |
| Opt. TNH – Run 1 | 59 | 27 | 23 |
| Opt. TNH – Run 2 | 22 | 15 | 10 |


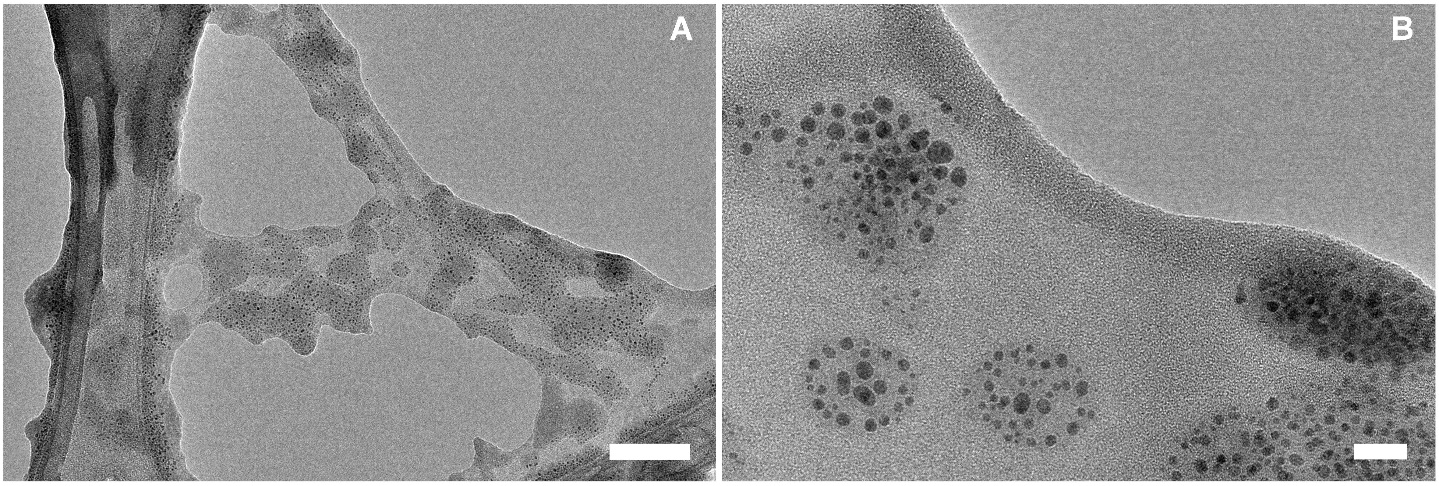


**Fig. S2. TEM images of the TNHs at 200 kV.** TEM of the freshly-prepared and just air-drying  TNH samples. The high acceleration voltage of the electron beam tends to rapidly melt the organic part, i.e. the EVs, leaving the ZnO NCs accumulated as they were in the round-shaped vesicles. (**A**) scale bar is 100 nm, (**B**) scale bar is 20 nm.
